# Neutrophil Extracellular Traps Are Pathogenic in Ventilator-Induced Lung Injury and Partially Dependent on TLR4

**DOI:** 10.1155/2017/8272504

**Published:** 2017-12-13

**Authors:** Haosi Li, Pinhua Pan, Xiaoli Su, Shuai Liu, Lemeng Zhang, Dongdong Wu, Haitao Li, Minhui Dai, Yi Li, Chengping Hu, Jie Chen

**Affiliations:** ^1^Department of Pulmonary and Critical Care Medicine, Xiangya Hospital, Central South University, Changsha, Hunan 410008, China; ^2^Department of Thoracic Medicine, Hunan Cancer Hospital and The Affiliated Cancer Hospital of Xiangya School of Medicine, Central South University, Changsha, Hunan 410013, China

## Abstract

The pathogenesis of ventilator-induced lung injury (VILI) is associated with neutrophils. Neutrophils release neutrophil extracellular traps (NETs), which are composed of DNA and granular proteins. However, the role of NETs in VILI remains incompletely understood. Normal saline and deoxyribonuclease (DNase) were used to study the role of NETs in VILI. To further determine the role of Toll-like receptor 4 (TLR4) in NETosis, we evaluated the lung injury and NET formation in TLR4 knockout mice and wild-type mice that were mechanically ventilated. Some measures of lung injury and the NETs markers were significantly increased in the VILI group. DNase treatment markedly reduced NETs markers and lung injury. After high-tidal mechanical ventilation, the NETs markers in the TLR4 KO mice were significantly lower than in the WT mice. These data suggest that NETs are generated in VILI and pathogenic in a mouse model of VILI, and their formation is partially dependent on TLR4.

## 1. Introduction

Acute respiratory distress syndrome (ARDS) is a life-threatening disease with a substantial mortality rate of 30–60% [1]. ARDS is also a common and costly public health problem, which poses an expensive burden to the entire world. One of the most important supporting therapies for ARDS patients is mechanical ventilation. However, this type of ventilation may further aggravate the lung injury, which is called ventilator-induced lung injury (VILI). Ventilator-induced lung injury can lead to high morbidity and mortality in these patients. Although the mechanisms of the development of VILI remain elusive, many studies have suggested that inflammatory mediators and cells are involved in the progress of VILI [2–4]. Activated neutrophils are among these mediators and are a key factor that contribute to the development of VILI, as confirmed by several studies [5, 6]. Neutrophil depletion in rabbits protects these animals against lung injury after mechanical ventilation [7].

In 2004, a new effector function of neutrophil, the so-called neutrophil extracellular traps (NETs), was discovered [8]. This process, which stimulates the neutrophils to release the NETs, is called NETosis [9]. NETs have a web-like structure and decondensed chromatin fibers (DNA) comprise its major structural component [10]. This web is decorated with granule proteins, such as elastase, myeloperoxidase, and histones [10]. Moreover, NETs also play an important role in immune defense. The main function of NETs is trapping and eliminating various types of invading pathogens, including bacteria, fungi, virus, and protozoan parasites [11–14]. Although NETs are helpful in infectious disease due to their antipathogen effects, their imbalance between production and clearance causes some negative effects to the hosts. This even exacerbates the progression of autoimmune and inflammatory diseases, including systemic lupus erythematosus, autoimmune small-vessel vasculitis, transfusion-related acute lung injury, and sepsis [15–18]. A recent study reported the cytotoxicity of epithelial and endothelial cells induced by NETs independently, not the other inflammatory components of neutrophils [19]. More recently, literature has emerged that offers contradictory findings about the role of NETs in the pathogenesis of VILI in mice. Rossaint et al. have shown that NETs directly influence the severity of VILI and contribute to the disease process [20]. In contrast to those findings, Yildiz et al. illustrate that NETs are formed in VILI but may have only a limited role in pathogenesis in the current model of VILI [21]. In that context, the role of NETs in the development of ventilator-induced lung injury remains to be elucidated.

Toll-like receptors (TLRs), serving as pattern recognition receptors (PRRs), are capable of recognizing various pathogens, including so-called pathogen associated molecular patterns (PAMPs), which mediate innate immune responses for host defence [22]. However, TLRs can be activated by the endogenous damage associated molecular patterns (DAMPs) that are released from damaged tissue or stressed cells which, thereby, induce sterile inflammation [23]. TLR4 is one of the most extensively studied TLRs and plays a fundamental role in the TLR family. TLR4 is involved in the inflammatory response of VILI [24–26], and the TLR4-MyD88 signaling pathway plays a key role in the pathogenesis of VILI [25, 27]. Additionally, TLR4 is a crucial regulator of neutrophil activation and survival [28]. However, it is unclear whether TLR4 is required for the formation of neutrophil extracellular traps in the context of VILI.

We hypothesized that NETs would be generated in a VILI mouse model and NETs could exacerbate lung injury. TLR4 would play a role in NET formation (NETosis). To test these hypotheses, we presented a mouse model of lung injury induced by high-tidal volume. Then the mice were treated with deoxyribonuclease (DNase) I, which destroyed the NETs, to study the role of the NETs in VILI. We targeted TLR4 using wild-type (WT) and TLR4 knockout (KO) mice.

## 2. Materials and Methods

### 2.1. Animals

TLR4 wild-type (C57BL/6J) mice and TLR4 knockout (KO) mice were purchased from the Jackson Laboratory (Bar Harbor, ME, USA). All the mice were male, 6–10 weeks old, and weighed 20 g to 25 g. The mice were maintained in the laboratory animal centre of the Central South University with specific pathogen-free (SPF) conditions. It also has controlled temperature, humidity, 12-hour light/dark cycle, and independent ventilation environment. All the procedures were approved by the Animal Care and Use Committee of the Central South University. They also were in accordance with the National Institutes of Health Guide for the Care and Use of Laboratory Animals. All the surgeries were performed under sodium pentobarbital anesthesia, and all efforts were made to minimize suffering.

### 2.2. Experimental Design

In the first set of experiments, the mice were randomized into the following three groups: (1) the control group (CON, *n* = 10); (2) the low-tidal volume ventilation group (LTV, *n* = 10); and (3) the high-tidal volume ventilation group (HTV, *n* = 10). In the second experiment, the mice were randomly assigned to two groups, the vehicle group (*n* = 10) and the DNase group (*n* = 10). In the third set of experiments, the WT mice and the TLR4 KO mice were investigated after mechanical ventilation. Therefore, the specimens were harvested from the WT (TLR4 WT CON, TLR4 WT LTV, TLR4 WT HTV groups, *n* = 10) and the TLR4 KO mice (TLR4 KO CON, TLR4 KO LTV, TLR4 KO HTV groups, *n* = 10).

### 2.3. Mechanical Ventilation in Mice

All the animals were anesthetized with sodium pentobarbital (50 mg/kg body weight) intraperitoneally and were placed in a supine position on a heating blanket to maintain a temperature of 37 ± 1°C. The anterior neck soft tissue was dissected under sterile conditions to expose the trachea. Then, the endotracheal intubation was performed with a 20-gauge intravenous (i.v.) catheter (B. Braun, Germany), and the catheter was connected to a small rodent ventilator (VentElite, Harvard Apparatus, Holliston, MA, USA) using room air and a volume-controlled setting. The following settings were used during protective ventilation and injurious ventilation: 7 ml/kg body weight with 135 breaths/min, 2 cm H_2_O positive end-expiratory pressure (PEEP) for 4 h; or 24 ml/kg with 100 breaths/min, 0 PEEP for 4 h. The control mice underwent a sham surgery and the endotracheal intubation but breathed spontaneously. For sham surgery, the mice's tracheas were exposed through an anterior neck soft tissue dissection under sterile conditions. After 4 hours, all the animals were given a lethal dose of the anaesthetic agent.

### 2.4. DNase I Treatment

These animals were instilled with 25 *μ*l of 3 mg/ml DNase I (Roche Diagnostics, Indianapolis, USA) or vehicle control (normal saline) twice through a 20-gauge catheter, followed by 200 *μ*l of air several times immediately. All the instillations were performed immediately after intubation and were then followed by mechanical ventilation with a high-tidal volume as described above.

### 2.5. Lung Wet/Dry Weight Ratio

The lung wet-to-dry weight ratio was analyzed as an index of pulmonary edema. After the mice were sacrificed, a portion of the right lung was excised and weighed for determination of the wet weight immediately. The lung weight was measured again after drying in an oven at 65°C for 72 h (dry weight).

### 2.6. Bronchoalveolar Lavage Analysis

Lung inflammation was evaluated by the concentration of protein and neutrophil counts in bronchoalveolar lavage fluid (BALF). The BALF procedure was performed with an instillation of sterile normal saline in a 0.5 ml volume with three replicates. More than 80% of the infused fluid was retrieved. All the samples were kept on ice until processed. The BALF samples were centrifuged (10 min, 1500 rpm, 4°C). The supernatants were removed and stored at −80°C. The pellet was resuspended in PBS, and the total cell numbers in the BALF were determined with a hemocytometer. Subsequently, the differential counts were performed on cytosine preparations stained with a modified Giemsa stain, Diff-Quick (Dade Behring AG, Düdingen, Switzerland).

### 2.7. Protein and Cytokines in BALF

The total protein concentration in the BALF was measured by using a BCA Protein Assay kit (Biomiga, USA) according to the manufacturer's instructions. Tumour necrosis factor-alpha (TNF-*α*) and interleukin-6 (IL-6) in the BALF were analyzed by using a mouse ELISA kit (RayBiotech, USA) according to the manufacturer's instructions. BALF level of high-mobility group box 1 protein (HMGB1) were detected by ELISA kit (Cusabio, China) according to the manufacturer's protocol.

### 2.8. Lung Histopathology

A portion of the left lung was removed and was fixed in 4% paraformaldehyde. After fixation, the lungs were dehydrated and embedded in paraffin. The sections (4 *μ*m) were stained with hematoxylin and eosin, and a histopathological analysis was performed by a pathologist who was blinded to the experimental protocol. The severity of VILI was evaluated according to the following items: alveolar congestion, hemorrhage, infiltration of neutrophils, and the thickness of the alveolar wall. Each item was scored on a scale of 0 to 4, as follows: 0, normal lungs; 1, mild damage, <25% lung involvement; 2, moderate damage, 25–50% lung involvement; 3, severe damage, 50–75% lung involvement; and 4, very severe damage, >75% lung involvement. The overall score of VILI is the sum of four items [29].

### 2.9. Immunofluorescence Staining of the Lung Sections

The lung tissue sections were fixed, stained, and imaged using confocal scanning laser microscopy. The lung tissue was incubated with the specific primary antibodies anti-citrullinated-histone H3 (1 : 100, Abcam, UK) or antineutrophil elastase (1 : 50, Santa Cruz Biotechnology, USA) overnight at 4°C. Primary antibodies were detected by Alexa Fluor 488 donkey anti-rabbit (1 : 500, Abcam, UK) and Alexa Fluor 647 donkey anti-goat (1 : 500, Abcam, UK) secondary antibodies. All the slides were visualized with an Olympus Fluoview 500 confocal laser scanning microscope.

### 2.10. Transmission Electron Microscopy

The lung tissue was excised, fixed with 2.5% glutaraldehyde, and then stored at 4°C for more than 24 h. The tissue was post-fixed in 1% osmium tetroxide in sodium cacodylate buffer, dehydrated using a graded acetone series, and then immerged in the solution of epoxy resin and acetone (1 : 1) for 24 h. Finally, the samples were embedded in Epon812, DDSA, MNA, and DMP30 at 60°C for 24 h. Ultrathin sections of 500 Egyptian thickness were cut on an LKB III microtome (LKB, Stockholm, Sweden). The sections were double-stained with uranyl acetate and lead nitrate. Subsequently, the sections were observed in a HITACHI-HT 7700 (Hitachi High-Tech, Tokyo, Japan) transmission electron microscope.

### 2.11. Western Blot

The lung homogenates were sonicated three times for 5 seconds each and were centrifuged at 15,000 g for 20 min at 4°C. The protein concentration in the supernatants was determined using the BCA Protein Assay kit (Biomiga, USA). The samples were resuspended in loading buffer and boiled for 5 min. Equal amounts of the proteins were resolved by electrophoresis using 12% sodium dodecyl sulfate-polyacrylamide (SDS-PAGE) gels and then transferred to polyvinylidene fluoride (PVDF) membranes (Bio-Rad Laboratories, Berkeley, USA). The membranes were blocked with 5% nonfat dry milk in TBS-T for 1 h at room temperature and were incubated overnight at 4°C with a primary antibody against citrullinated histone-3 (1 : 1000, Abcam, UK) and GAPDH (1 : 2000, Proteintech, USA) as an internal control. This incubation was then followed by incubation with a secondary antibody conjugated with horseradish peroxidase (HRP) at room temperature for 1 h. The membranes were visualized using an enhanced chemiluminescence (ECL) Western blotting detection reagent (Advansta, Melo Park, USA) and were exposed to film. The quantification was performed with Image J software (V.1.5, National Institutes of Health, USA).

### 2.12. Quantifying BALF DNA

The bronchoalveolar lavage fluid cell-free DNA was quantified by Quant-iT™ PicoGreen® (Invitrogen, Canada) following the manufacturer's protocol.

### 2.13. Statistical Analysis

The results are presented as the mean ± standard deviation (SD). The data were analyzed by SPSS Version 22.0 software. Two groups comparisons were analyzed using Student's *t-*test. Three groups comparisons were analysed using one-way ANOVA and Tukey post hoc test or Dunnett T3 test. Six groups comparisons were performed using a two-way ANOVA. ANOVA was followed by Tukey post hoc test when significant effects were found. The threshold of a *P* value less than 0.05 was considered statistically significant.

## 3. Results

### 3.1. Formation of Neutrophil Extracellular Traps in VILI

**Figure 1:**
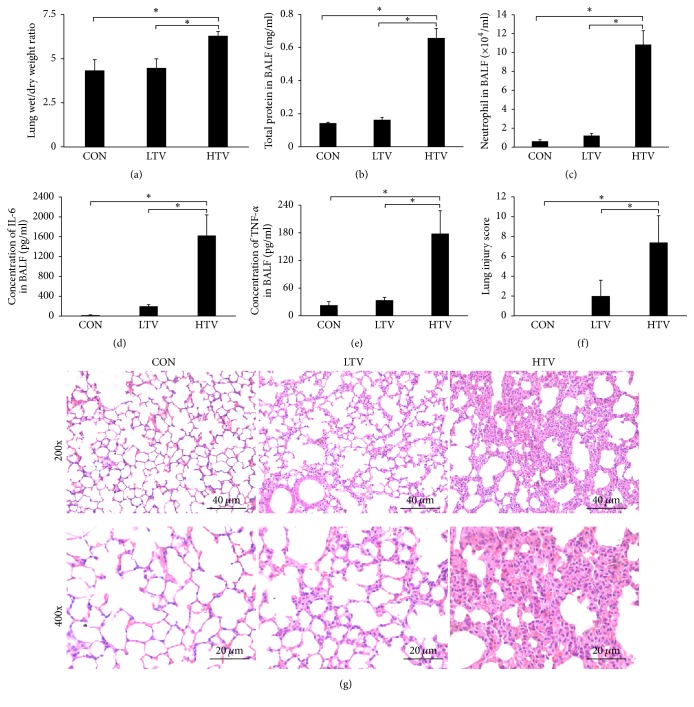
Ventilation with high-tidal volume causes lung injury. (a) The lung wet-to-dry weight ratio was increased in the HTV group compared with the CON group and the LTV group. (b) The concentration of the total protein in the BALF was significantly elevated in the HTV mice compared to the CON mice and the LTV mice. (c) The number of neutrophils in the BALF was increased in the HTV group. (d)(e) The BALF levels of IL-6 and TNF-*α* were markedly elevated in the HTV mice compared with the two other groups. (f) The lung injury score in the HTV group was markedly elevated compared to the other groups. (g) Low (magnification 200x, scale bar = 40 *μ*m) and high power (magnification 400x, scale bar = 20 *μ*m) views of the lungs of mice subjected to spontaneous ventilation, protective ventilation, and injurious ventilation. Hematoxylin and eosin stain. CON group: there are no obvious histological changes in the control group; the HTV group mice have much more inflammatory cell infiltration, alveolar septal thickening, pulmonary edema, and hemorrhage than in the CON and LTV groups. All the data are presented as the mean ± SD. ^*∗*^*P* < 0.05.

We compared the following three groups of mice: the spontaneous ventilation groups (CON), the low-tidal volume (LTV) ventilation group, and the high-tidal volume (HTV) ventilation group. As illustrated in Figure 1(a), the lung wet/dry weight ratio was increased in the HTV group compared with the CON group and the LTV group. Compared to the CON mice and the LTV mice, the BALF total protein concentration and the neutrophil count in the BALF were significantly elevated in the HTV mice (Figures 1(b)-1(c)). In addition, in the HTV mice, the BALF levels of IL-6 and TNF-*α* were elevated markedly compared with the two other groups (Figures 1(d)-1(e)).  Figure 1(g) shows representative optical microscopic lung histopathological findings, which revealed that the CON mice displayed normal lung histology without any inflammation, and the LTV mice showed a mild level of lung tissue damage. Furthermore, there was significant inflammatory infiltration, alveolar septal thickening, pulmonary edema, and hemorrhage in the HTV group, compared with the two other groups. The lung injury score in the HTV group was also markedly elevated compared to the other groups (Figure 1(f)).

**Figure 2:**
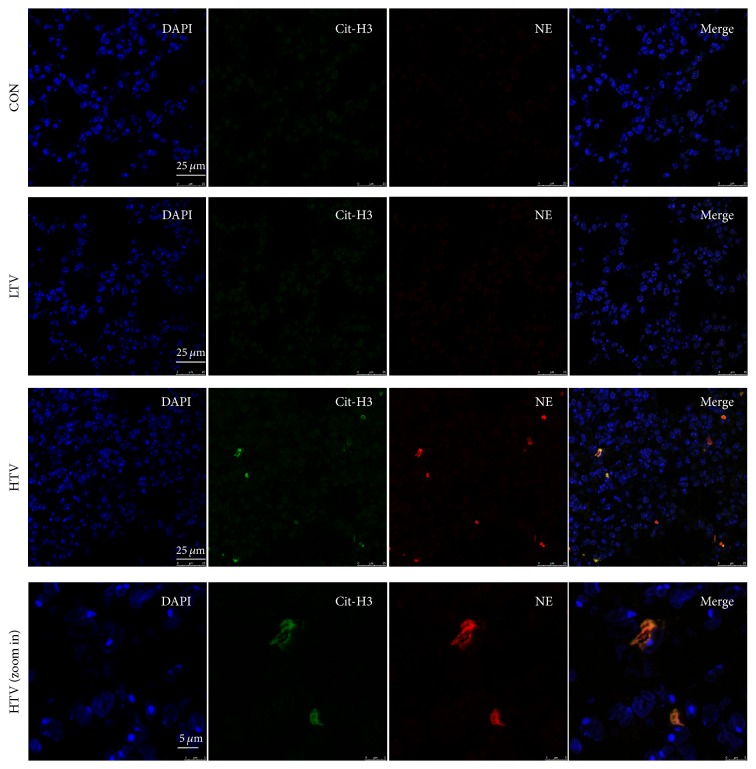
Immunofluorescence staining of Cit-H3 and NE on lung sections. NETs, defined as colocalized DNA (blue), citrullinated histone-3 (Cit-H3) (green), and neutrophil elastase (NE) (red), presented in lungs sections of VILI model (HTV). However, there was no obvious staining in CON group and LTV group.

**Figure 3:**
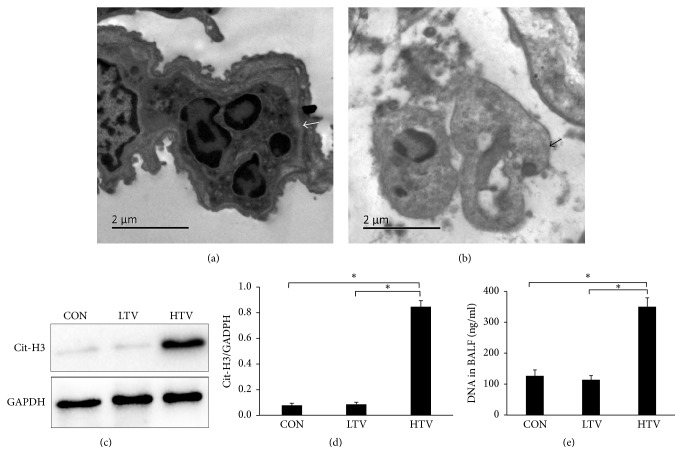
NETs formed in the lung of the VILI animal model. (a) Normal neutrophils in the airway with an intact nuclear membrane, located in the alveolar space, are shown by transmission electron micrograph (as indicated by the white arrows). Scale bar = 2 *μ*m. (b) Neutrophils with a collapse of the nuclear membrane, dispersed chromatin, and nuclear vesicles (as indicated by the black arrows) in the alveolar space. Scale bar = 2 *μ*m. (c) Representative in the lung homogenates. All the experiments were repeated at least 3 times. Only the HTV group has a strong Cit-H3 band. (d) The ratio of the Cit-H3/GAPDH intensity is significantly higher in the HTV group compared to the other groups. (e) The HTV mice produced a remarkably high level of BALF free DNA compared to the CON and LTV groups. ^*∗*^*P* < 0.05.

To investigate whether the NET formation will occur in VILI, citrullinated histone-3 (Cit-H3), neutrophil elastase (NE), and DNA, which are known as markers of NET formation, were identified by immunofluorescence staining. The colocalized Cit-H3, NE, and DNA staining presented in the HTV mice instead of in the CON and LTV mice (Figures 2(a)–2(c)). To further confirm the NET formation, the lung sections from the mice were observed by transmission electron microscopy (TEM). The TEM results revealed some normal neutrophils with an intact nuclear membrane located in the alveolar space (Figure 3(a)). Neutrophils with a collapse of the nuclear membrane, dispersed chromatin, and nuclear vesicles located in the alveolar space were also shown by TEM (Figure 3(b)), which is known as one of the steps of NET formation. We also analyzed the expression of Cit-H3 in the lung homogenates by Western blot to quantify the NET formation. There was a high level of Cit-H3 only in the HTV group (Figure 3(c)), which demonstrated that Cit-H3 was induced in VILI. The ratio of Cit-H3/GAPDH intensity is also significantly higher in the HTV group compared to the other groups (Figure 3(d)). We then measured the levels of free DNA in the BALF. The HTV mice produced a remarkably high level of BALF free DNA compared to the CON and LTV groups (Figure 3(e)).

### 3.2. DNase I Reduces NET Formation and Attenuates Lung Injury

**Figure 4:**
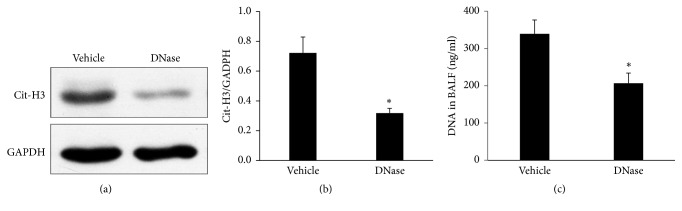
DNase I treatment abolished NET formation in vivo. (a) The DNase I pretreated mice had a significantly decreased level of Cit-H3 expression in the lung homogenates by a Western analysis compared to the vehicle group. (b) The ratio of the Cit-H3/GAPDH intensity was significantly lower in the DNase group compared to the vehicle groups. (c) The DNase treated mice produced a lower level of BALF DNA compared to the vehicle group. ^*∗*^*P* < 0.05 compared to the vehicle group.

Although neutrophil extracellular traps play an important role in inflammatory diseases, a role in VILI has not been described clearly. To address this question, one group of animals were instilled intratracheally with DNase I, which degrades NETs, or with vehicle, as a control, prior to the ventilation. Then, the NET formation and the lung injury were detected. The DNase I pretreated mice had a significantly decreased level of Cit-H3 expression in the lung homogenates by a Western analysis compared to the vehicle group (Figure 4(a)). The ratio of the Cit-H3/GAPDH intensity was remarkably lower in the DNase group compared to the vehicle groups (Figure 4(b)). Furthermore, the DNase group exhibited relatively lower levels of BALF free DNA than the vehicle group (Figure 4(c)).

**Figure 5:**
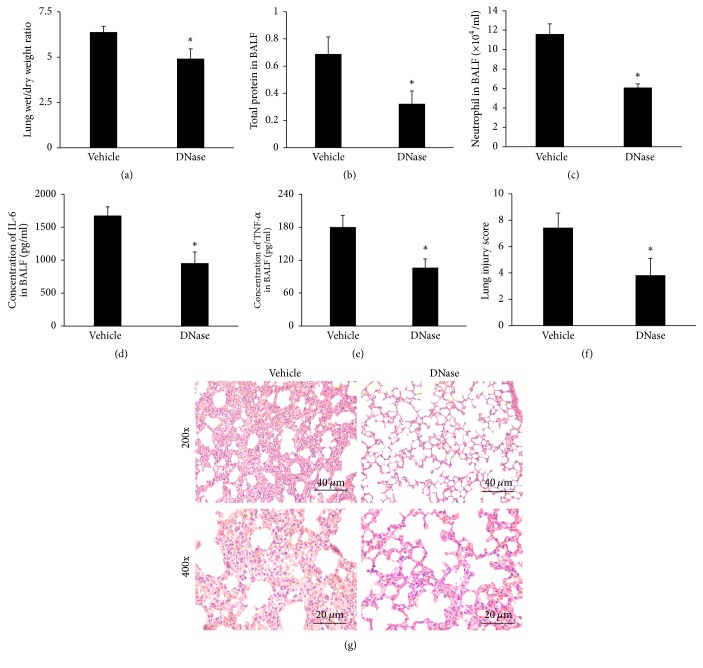
DNase attenuates lung injury and inflammation in mice. (a) The lung wet/dry weight ratio decreased partially in the DNase group compared with the vehicle group. (b)(c) The BALF total protein concentration and the neutrophil counts in the BALF all decreased in the DNase I pretreated mice (versus vehicle control). (d)(e) DNase I also reduced the BALF levels of IL-6 and TNF-*α*. (f) The lung injury score in the vehicle group was markedly higher than those in the DNase group. (g) Low (magnification 200x, scale bar = 40 *μ*m) and high power (magnification 400x, scale bar = 20 *μ*m) views of the lungs of the vehicle group and the DNase group. Hematoxylin and eosin stain. The vehicle group had much more inflammatory cell infiltration, alveolar septal thickening, pulmonary edema, and hemorrhage than the DNase group. ^*∗*^*P* < 0.05 compared to the vehicle group.

In addition, the lung wet/dry ratio declined partially in the DNase I pretreated group compared with the vehicle group (Figure 5(a)). Likewise, the BALF total protein concentration and the neutrophil counts in the BALF were all decreased in the DNase I pretreated mice (versus vehicle control) (Figures 5(b)-5(c)). DNase also reduced the BALF levels of IL-6 and TNF-*α* (Figures 5(d)-5(e)). In addition, as illustrated in Figure 5(g), the DNase group exhibited an alleviated lung injury under optical microscopy, and the lung injury score in the DNase group was also markedly lower than in the vehicle group (Figure 5(f)).

### 3.3. TLR4 Is Involved in NET Formation in VILI

**Figure 6:**
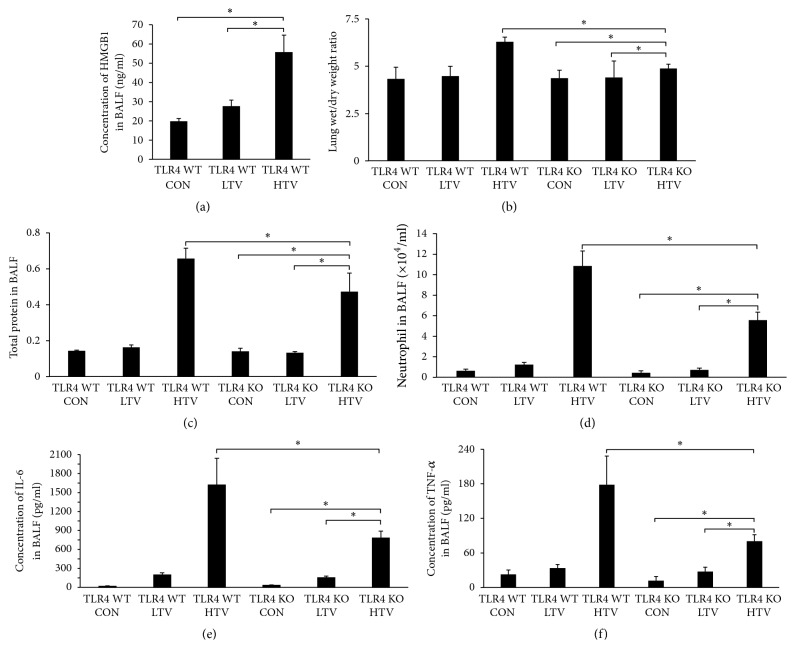
Toll-like receptor 4 knockout prevents the development of lung injury (A). (a) The level of HMGB1 in BALF was significantly higher than that in TLR4 WT CON group and LTV group. (b) The lung wet/dry ratio in TLR4 KO HTV mice was decreased compared with the TLR4 WT mice following HTV. (c)(d) The TLR4 KO HTV mice showed a marked elevation in protein content and neutrophil counts in the BALF compared to the TLR4 WT HTV mice. (e)(f) The BALF levels of IL-6 and TNF-*α* in the TLR4 KO HTV mice did not increase as much as in the TLR4 WT HTV mice. From (b) to (f), two-way ANOVA indicated both TLR4 genotype (TLR4 WT versus KO) and ventilation were significant, but there was no interaction (*P* < 0.05). ^*∗*^*P* < 0.05.

**Figure 7:**
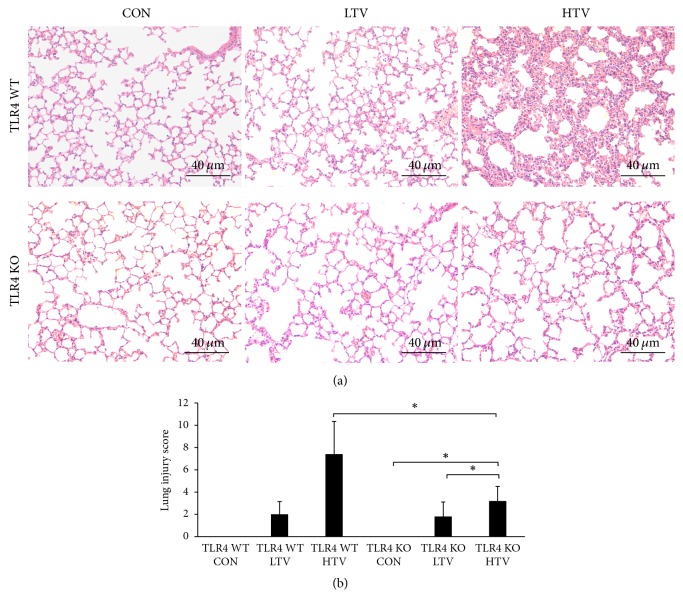
Toll-like receptor 4 knockout prevents the development of lung injury (B). (a) Low (magnification 200x, scale bar = 40 *μ*m) power views of the lungs of spontaneous ventilation, protective ventilation, and injurious ventilation in the TLR4 WT mice and the TLR4 KO mice, respectively. Hematoxylin and eosin stain. The TLR4 KO mice have less inflammatory cell infiltration, alveolar septal thickening, pulmonary edema, and hemorrhage than the TLR4 WT mice during HTV. (b) The lung injury score in the TLR4 KO HTV group was significantly lower than those in the TLR4 WT HTV group. Two-way ANOVA indicated that only ventilation was significant (P>0.05), but analyzing TLR4 WT HTV and TLR4 KO HTV with Student's* t*-test indicated a statistical significance (*P* > 0.05). ^*∗*^*P* < 0.05.

Based on the previous results, TLR4 plays a key role in the pathology of VILI in mice. Therefore, we assumed that TLR4 contributes to NET formation in VILI in mice, and we hypothesized that HMGB1 is involved in TLR4 activation. As shown in Figure 6(a), the level of HMGB1 was vastly higher than that in TLR4 WT CON group and LTV group. The lung wet/dry ratio was relatively increased in the TLR4 KO HTV mice compared with the TLR4 KO LTV mice and the TLR4 KO CON mice (Figure 6(b)). But it was decreased compared with the TLR4 WT mice following HTV (Figure 6(b)). The TLR4 KO HTV mice showed a marked reduction of protein content and neutrophil count in the BALF compared to the TLR4 WT HTV mice (Figures 6(c)-6(d)). Similarly, the BALF levels of IL-6 and TNF-*α* in the TLR4 KO HTV mice did not increase as much as those in the TLR4 WT HTV mice (Figures 6(e)-6(f)). The optical microscopic lung histopathological findings and the lung injury score indicate that the TLR4 KO HTV mice developed less lung injury than the TLR4 WT HTV mice (Figures 7(a)-7(b)).

**Figure 8:**
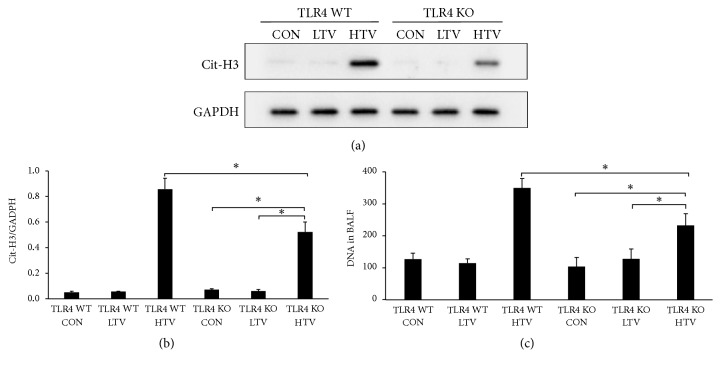
Toll-like receptor 4 contributes to the NET formation in VILI in mice. (a) In contrast to the TLR4 WT mice, HTV partially increased Cit-H3 expression in the lung homogenates in the TLR4 KO mice. (b) The ratio of the Cit-H3/GAPDH intensity in the TLR4 KO HTV group was lower compared to the TLR4 WT HTV mice. Two-way ANOVA indicated that only ventilation was significant (P < 0.05), but analyzing TLR4 WT HTV and TLR4 KO HTV with Student's* t*-test indicated a statistical significance (P < 0.05). (c) The release of DNA in the BALF was markedly decreased in the TLR4 KO mice compared with the TLR4 WT mice after 4 h of ventilation. Two-way ANOVA indicated both TLR4 genotype and ventilation were significant, but there was no interaction (*P* < 0.05). ^*∗*^*P* < 0.05.

Since the above results showed that the lung injury following HTV was significantly attenuated in the TLR4 KO mice, we next assessed whether TLR4 influences NET formation in VILI mice model. We found that, in the TLR4 WT HTV mice, the expression of Cit-H3 in the lung homogenates increased significantly compared with the CON and LTV groups. In contrast to the TLR4 WT mice, HTV partially increased Cit-H3 expression in lung homogenates in the TLR4 KO mice (Figure 8(a)). The ratio of the Cit-H3/GAPDH intensity in the TLR4 KO HTV group was lower than the TLR4 WT HTV mice (Figure 8(b)). Furthermore, the release of DNA in the BALF was markedly decreased in the TLR4 KO mice compared with the TLR4 WT mice after 4 h of ventilation (Figure 8(c)).

## 4. Discussion

In this study, we illustrated the following: ventilation with high-tidal volume causes lung injury and inflammation in mice; NETs increase in the lung of the VILI animal model upon the elevation of known NET-associated proteins and extracellular DNA; pretreatment of NET degradation with DNase I alleviates the preceding lung injury, which means that NET formation has an adverse effect on the development of VILI; and TLR4 is involved in NET formation in VILI in mice.

To test the hypothesis of the study, we successfully established a mouse model for VILI, following the ATS criteria for experimental acute lung injury in animals [30], with 24 ml/kg body weight × 100 breaths/min × 4 h × 0 PEEP. The current results demonstrated that high-tidal volume caused lung injury, which included increased wet-to-dry weight ratios, high histopathological scores, raised pulmonary microvascular permeability with the extravasation of plasma proteins, and polymorphonuclear leucocyte accumulation. Last but not least, the levels of proinflammatory cytokines, such as IL-6 and TNF-*α*, were elevated. Although the high-tidal volume (24 ml/kg) in this study is much higher than that in the clinical setting, it is reported that low-tidal volume ventilation also causes regional lung overdistension and lung injury in some patients, especially ARDS patients [31, 32]. Furthermore, our experimental animals were healthy mice with the previously unaffected lung. We must use the high-tidal volume intentionally to induce acute lung injury, which is helpful to study the effects of purely mechanical stress. Additionally, the setting in this study is similar to that in other research of VILI, which makes the results of our article more comparable.

Our study identified that NETs increase in the lung of a VILI animal model. We found the colocalization of DNA, Cit-H3, and NE in the lung, which was caused by lung injury induction of HTV. Moreover, it also caused level increments of Cit-H3 in the lung homogenates as well as the DNA in BALF. However, extracellular DNA in BALF may also derive from necrotic cells, and neutrophils are one of the main cells which die in the airways. We supported our findings by detecting Cit-H3 because it was proved to be vital in NET formation [33] and also a widely used marker for NETs.

A previous study concluded that DNase I, instead of proteases, degrades NETs [34]. Our study also found that the Cit-H3 level in the lung homogenates and the levels of DNA in the BALF were both notably decreased in the VILI mice model with preinstilled DNase I, suggesting that DNase I treatment is an effective way to reduce NET formation in a VILI mouse model. Our present experiments have shown the reduction of all the measures of lung injury of DNase I-treated VILI mice. Thus, our study suggested that DNase pretreatment partially attenuates lung injury induced by high-tidal volume, and NETs could constitute a role in the pathogenesis of VILI. There are some possible explanations for why the DNase cannot entirely recover the lung injury. The pathogenesis of VILI is complicated and involves multiple pathways, including alveolar macrophages activation, neutrophils infiltration, and the release of various inflammatory mediators [35, 36]. NETs are the only one part in a complex series of events. Moreover, DNase cannot degrade NET components completely in vivo.

Although the mechanism of aggravation of ventilator-induced lung injury by NETs is not thoroughly understood, it may exert a detrimental effect on the lungs through these processes. The DNA fibres, closely entangled to the alveolar epithelium, appear to be harmful to the alveolar-capillary barrier [37], and the leakage of nucleic acids potentially damages tissue, which play a role in development of autoimmune diseases [10]. Furthermore, some proteins in the NET web may lead to tissue damage. For example, histones and myeloperoxidase directly influence the host cell, which are closely related to NET-mediated cytotoxicity [19]. Neutrophil elastase also contributes to tissue damage [38].

Our results are in agreement with Rossaint et al.'s findings which showed that modulating NETs by DNase I influences the severity of VILI [20]. We both used single-hit animal model (injurious ventilation alone). However, Yildiz et al. showed that DNase treatment cannot significantly protect some measures of lung injury [21]. These differ from the findings presented here. There are several possible explanations for this inconsistency. First, Yildiz et al. established a two-hit lipopolysaccharide/VILI model. The pathogenesis of preexisting inflammatory stimulus (e.g., lipopolysaccharide) might be different from that of single physical stimulus (e.g., injurious ventilation). For example, the lungs of two-hit mice model were already filled with neutrophils before the progress of VILI due to LPS instillation. This might be difficult to attenuate by DNase. Second, the concentration of DNase is different. Our animals were instilled with 25 *μ*l of 3 mg/ml DNase I twice. However, mice received the same volume of 1 mg/ml DNase twice in Yildiz et al.'s study.

Some studies have suggested that the high-mobility group box 1 (HMGB1), which can be secreted by necrotic cells or inflammatory cells, specifically acts through multiple cell surface receptors, including TLR4 and TLR2. Moreover, an* in vitro* study showed that the TLR4-mediating HMGB1 promotes NET formation [39]. Consistent with the expectation, TLR4 WT HTV mice had a high level of HMGB1, which suggested that how TLR4 is activated. In this study, we showed the difference in the results of the independent experiments between the TLR4 KO mice and wild-type mice following high-tidal volume, which clearly supported the effect of the TLR4 on NET formation in the VILI model. Some previous studies have demonstrated that TLR4 plays an essential role in NET generation. For example, the neutrophils isolated from TLR4^−/−^ mice showed decreased levels of NET formation after stimulation by nontypeable* Haemophilus influenzae* (NTHi) [40]. In addition, the RSV F protein is capable of inducing NET release through TLR4 activation [41]. Our study demonstrated, for the first time, that TLR4 is required for NET formation in the VILI model.

However, there was still a certain level of NETs formation in TLR4 KO HTV mice. Therefore, other pathways may also be involved in NETs stimulation. Some studies have shown that various pathways, including TLR2 [42–44], TLR7, TLR8 [45], TLR9 [46], and RAGE [47], can mediate NET formation. For instance, RAGE knockout animals had lower levels of serum DNA and decreased citrullinated histone H3 expression [47]. Moreover, Saitoh et al. found that NET formation can be activated by human immunodeficiency virus 1 (HIV­1), probably through TLR7 and TLR8 [13].

Additionally, TLR4 KO protects mice from lung injury not only by inhibiting NET formation but possibly also by other mechanisms. For example, histones have been identified as DAMP molecules or alarmins during tissue injury. It has been shown that histones specifically bind to and activate TLR4, and histones mediated tissue injury is partially dependent on TLR4 [48, 49]. Xu et al. reported that extracellular histones induce cytokine production in vivo primarily in a TLR4-dependent manner.

In conclusion, our present findings suggest that NETs increase in the lung of a VILI animal model and are implicated in the pathogenesis of VILI. Furthermore, NET formation is partially dependent on TLR4. Thus, the therapeutic inhibition of NET formation may offer a promising new approach for patients suffering from VILI.
